# Understanding Digital Inequality: A Theoretical Kaleidoscope

**DOI:** 10.1007/s42438-023-00395-8

**Published:** 2023-03-23

**Authors:** Caroline Kuhn, Su-Ming Khoo, Laura Czerniewicz, Warren Lilley, Swati Bute, Aisling Crean, Sandra Abegglen, Tom Burns, Sandra Sinfield, Petar Jandrić, Jeremy Knox, Alison MacKenzie

**Affiliations:** 1 Bath Spa University, Bath, UK; 2 National University Ireland Galway, Galway, Ireland; 3 University of Cape Town, Cape Town, South Africa; 4 Jagran Lakecity University, Bhopal, India; 5 University of St., Andrews, St. Andrews, Scotland, UK; 6 University of Calgary, Calgary, Canada; 7 London Metropolitan University, London, UK; 8 Zagreb University of Applied Sciences, Zagreb, Croatia; 9 University of Edinburgh, Edinburgh, UK; 10 Queen’s University, Belfast, UK

**Keywords:** Theoretical kaleidoscope, Toolkit, Methodology, Digital inequalities, Postdigital, Collaborative writing

## Abstract

The pandemic affected more than 1.5 billion students and youth, and the most vulnerable learners were hit hardest, making digital inequality in educational settings impossible to overlook. Given this reality, we, all educators, came together to find ways to understand and address some of these inequalities. As a product of this collaboration, we propose a methodological toolkit: a theoretical kaleidoscope to examine and critique the constitutive elements and dimensions of digital inequalities. We argue that such a tool is helpful when a critical attitude to examine ‘the ideology of digitalism’, its concomitant inequalities, and the huge losses it entails for human flourishing seems urgent. In the paper, we describe different theoretical approaches that can be used for the kaleidoscope. We give relevant examples of each theory. We argue that the postdigital does not mean that the digital is over, rather that it has mutated into new power structures that are less evident but no less insidious as they continue to govern socio-technical infrastructures, geopolitics, and markets. In this sense, it is vital to find tools that allow us to shed light on such invisible and pervasive power structures and the consequences in the daily lives of so many.

## Introduction

Internet and Information and Communication Technologies (ICTs) have become ubiquitous in workplaces and homes, through either their visible existence or their invisible impact. The widespread existence of ICTs has given rise to what Castells (2001) and others (e.g. Van Dijk 2001: 3) call the network society: ‘an information society with a nervous system of social and media networks shaping its prime modes of organisation and more important structures’. For the human body, a healthy nervous system is critical for a fully functional life. Similarly, access to this ‘nervous system’ of social and media networks is paramount for individuals to be fully able to participate in the different realms of this networked society.

Unfortunately, it became evident during the Covid-19 pandemic how precarious the nervous system of the networked society is as more than a third of all students globally were unable to access education, detrimentally affecting their present and future life (UNESCO 2021; Jandrić et al. 2021b) and further reinforcing already well-known historical inequalities. Researchers recognised this reality early on, as a review of teaching and learning research during the first year of the pandemic found that inequality was a key focus of research interest (Stewart 2021).

### Why Does Digital Inequality Matter?

High levels of inequality negatively affect society as a whole, not just the less advantaged. More unequal societies have higher crime rates, weaker property rights, skewed access to social services, less influence on decision-makers, and slower transitions to democracy (Helsper 2021; Wilkinson and Pickett 2009). Individuals can only flourish if all other individuals are doing so. Thus, finding ways to address and alleviate the stark and ever-increasing digital inequality that, although not new, has been crudely exposed during the pandemic is vital to us all. What has been clear from multiple studies is that the links between technology and inequality are highly complex and multifaceted (Eynon 2022: 1); one could even argue, super complex (Barnett 2000a, b; Abegglen et al. 2020a).

Experiences and effects are diverse, multiple, and often contrasting, with frames of reference intersected by uncertainty, unpredictability, and fragility. Thus, we need to discuss not just the technology itself but the practices surrounding its use for teaching and learning. This includes taking a critical stance and questioning the structures and processes that facilitate/constrain students’ and educators’ ability to participate and take action. As Barnett (2000a, b) argues, the main pedagogical task of a university is not to transmit knowledge but to develop students’ attributes appropriate to the conditions of supercomplexity, and we add, to the conditions of the postdigital (Jandrić et al. 2018), which is to treat digital and human social life as fundamentally intertwined.

Historically, the supercomplex nature of digital inequality has been underplayed and under-theorised. Traditional accounts of digital inequality have centred on the lack of access to ICTs, framing the discussion in terms of the ‘haves’ and ‘have-nots’ (see Light 2001; van Dijk 1999, 2006; DiMaggio and Hargittai 2001), and have also concentrated on the technology as such, emphasising these devices as neutral within the contexts they are placed, overlooking the fact that they are impacted by greater socio-cultural constraints and users’ agency, as well as new articulations of uneven power relations.

In relation to the postdigital condition, it is important to note that the prefix ‘post’ has nothing to do with the digital being over, but that the digital has progressed from a discrete point of departure to an ongoing condition, a way of life everyone is part of, even the disconnected, who unknowingly contribute digital data to socio-technical infrastructures. Cramer (2015) believes, as we do, that the postdigital means that new power structures become less evident, but not less insidious as they continue to govern such socio-technical infrastructures as well as geopolitics, and markets. For this reason, it is vital to find tools that allow us to shed light on such invisible and pervasive power structures and the consequences of their exercise in the daily lives of so many.

Currently, governments, organisations, and individuals are wrestling with what digital inequality means in an increasingly digitalised, postdigital, and post-pandemic world and how they can confront it. Unfortunately, interventions guided by simplistic, uncritical, and apolitical accounts of digital inequality are more likely to entrench inequality than find pathways for equitable transformation, especially in education (see Lilley 2022).

### Why Different Theories to Understand Digital Inequalities?

Our starting point is that digital inequality is a complex phenomenon and that different theoretical approaches may help to diagnose different aspects of what is wrong and why. If an injustice is misdiagnosed, it can lead to strategies which may not only be ineffective, but potentially create further injustices. Guided by Lewin’s maxim that nothing is as practical as a good theory (McCain 2016), we believe it is important to elucidate what different approaches can be offered, which might be helpful to answer particular questions to address particular injustices but not others. Given that digital inequality is a supercomplex social phenomenon, stratified, and multidetermined, it is our contention that different ways of theorising can clarify a greater range of structural solutions to the social problem of inequities.

## A Theoretical Kaleidoscope

We use the metaphor of a kaleidoscope to describe the need for different theories, or sometimes, the intersection of multiple theories, to unpack and understand the complexity of digital inequality. When discussing the relationship between theory and research, ‘theoretical lenses’ are posited to help showcase how a particular theory provides specific concepts when examining any social phenomenon. Kaleidoscopes are different. This optical instrument uses two or more mirrors/lenses, angled at particular points, which, when rotated, allow the viewer to see an increasing array of complex patterns that would be hard to see with our naked eye. It is important to note that with each modest turn of the kaleidoscope, the image shifts slightly, offering a different perspective, colour, or intricacy. The kaleidoscopic image is shaped by the number of mirrors in the kaleidoscope.

The mirrors in the kaleidoscope represent the theories chosen by the researcher depending on her/his/their needs. While all the theories centre on the importance of recognising digital inequality as a supercomplex phenomenon, each view is angled at a distinct point, highlighting specific patterns or features for the researcher to discern. Just like a kaleidoscope, as the reader turns to a new theory or the intersection of many, we aim to support them to appreciate and understand the nuanced nature of digital inequality that the theory or the intersection of them unveils as well as its relationship to previously discussed theories. In short, what the kaleidoscope does is serve as an analytical tool to examine, critique, and understand different dimensions of digital inequality and in so doing, we hope that a variety of alternatives and novel solutions can be found to address the insidious consequences of digital inequalities.

For example, Eynon (2022) points out that in academic research, the relationships between individual Internet use and social opportunities are typically understood within the classic sociological problem of structure versus agency, pointing out that digital inclusion scholars have tended to privilege either structure or agency. When structure and agency are conflated, problems can seem circular and difficult to break open and understand. Similar to other work in this domain, it is clear that outcomes of Internet use should not only be understood as the product of access and skills, but it is crucial to attend to socio-cultural structural conditions (Eynon 2022).

Therefore, using a social theory that allows us to study the interplay of structure and agency (e.g. see the ‘Critical Realism and Realist Social Theory’ section) rather than conflating both or privileging one over the other will be useful to acknowledge the importance of both people’s actions–agency–and the role of social structure in constraining or enabling those actions. Other theories will focus more on the person in all their humanity–their strengths, frailties, hopes, and fears. For example, critical pedagogical theories can be used to look into more human dimensions of the phenomenon. They can, amongst other things, help develop an emancipatory ‘sociological imagination’ (Mills 2000).

### Why A Collaborative Piece?

Collective writing aims to organise diversity rather than replicate uniformity (Peters et al. 2021). Coming together to write seemed a positive, constructive way to approach digital inequality. Our conversation as a group of authors began in an international online event on digital inequality, followed by our mutual explanations regarding which theories we had each found useful in our own research and why. Through our shared experiences, it became obvious that inequality can be explored in different ways using different lenses, each with advantages and limitations. The joint unravelling of the complex nature of digital inequality created an energy, a collective generativity, of doing something together that was multifaceted.

Collaborative writing is a form of ‘resistance’ in itself. Greene (2007) considers it an approach that is sometimes deemed countercultural since the academic norm, particularly in the humanities, is the lone scholar, and the ‘gold standard’ writing product is the single-authored monograph. Collaborative writing has particular pragmatics and ethics: as a ‘coming together’, as an observational tool (Magnusson 2021), and as a method of inquiry (Gale and Bowstead 2013), pushing us towards a different understanding, a continuous struggle for meaning-making (Jandrić et al. 2022a, b). Starting from where we are, we acknowledge the problems as we generate a shared sense of, and hope for, higher education ‘otherwise’. By working with each other and creating an assemblage, the act of writing evokes something new, which provokes and touches–an emergent praxis of enquiry (Gale et al. 2012, 2014).

Collaborative writing goes beyond a simple, efficiency-driven division of labour (cooperative writing). It requires the co-authors to be involved in all stages of the writing process, sharing the responsibility for and the ownership of the entire text produced (Storch, 2019). The pragmatics begin the work of collaborative writing through call and response (being asked to write sections) and collective crafting (deciding who will do what, the order of things, weaving together). The ethics of collaboration emerges from the process, the situated experiences of trying to understand and appreciate what each contributor brings, to structure our contributions and seek responses. We consider, thus, it is a more ‘response-able’ approach to navigating the ethics of various facets of digital inequalities, respecting the many-sided character of the complex inequalities involved in its apprehension.

### A Brief Overview of the Theories Presented to Craft the Kaleidoscope

To answer the questions and concerns above, we assembled a group of theoretical approaches that can address various dimensions of digital inequality. We recognise that the theories discussed in this article, and summarised below, can only provide a partial picture. At the same time, these theories are tried and tested in our work, and those of many others, hence the sharing of these lenses. We begin with the *capability approach* (Sen 1992; Nussbaum 2011; Robeyns 2017), which is mindful of people’s differences by questioning what it means to offer equal access via digital means. In the capability approach, inequality is understood as unequal capabilities to do and be things that people have reasons to value. We follow with Bourdieu’s *theory of practice* (1972), where inequality is understood through the key concepts of field, habitus, and capital. For Bourdieu, inequality goes beyond a person’s goods and economic resources. Instead, it is linked to economic, cultural, and symbolic capital. Next, we touch on *cultural-historical activity theory* (CHAT) (Engeström 1987, 2011) which focuses on the socio-cultural structures and interdependent relationships between the individual and the community that enable and/or constrain the uptake of digital technologies.

*Affective injustice* (Srinivasan 2018; Whitney 2018) is another lens through which we might view our responses to the emotional lives of others as a distinctive source of social inequality and injustice. For example, due to status or stigmatising differences, the emotional lives of the less socially equal are given less weight than is appropriate. As a result, they are made to experience themselves as relationally inferior and are treated as such. For Jan van Dijk’s *resources appropriation theory*, the problem of digital inequality starts with how people use digital media in their daily lives. Personal and positional differences generate inequalities in the distribution of resources (e.g. income, social network, status), resulting in disparities in the process of technology appropriation. We also include *critical pedagogy* (Freire 1972/2018), an approach which problematises the notion that technology automatically grants access and enhances learning. Instead, educators and students need to learn how to harness digital education for liberatory purposes–for agency and ‘action’. By including the work of Fraser (2008a, b), we offer a *tripartite model of justice* that provides a broader understanding of what injustice is by adding two dimensions besides distribution–cultural injustice and political injustice. This tripartite model offers the researcher a lens to look into digital inequality decentred from technology that focuses on issues of misrecognition and misrepresentation in the digital world but also outside of it. The theory provides concepts and ideas to address injustices derived from institutionalised hierarchies of cultural value and misrepresentation of political voicelessness.

Our final kaleidoscopic lens offered is *critical realism* (CR) and *realist social theory* (RST), which work in tandem to address the ‘why’, ‘what’, and ‘under which circumstances’ questions in social science. These theories bring attention to the interplay of structure, culture, and agency in inequality, particularly in social reproduction/change, i.e. morphogenesis/morphostasis. For CR proponents, the critical question is how digital inequality is produced, reproduced, and transformed, and what mechanisms and actions interact to arrive at the problematic event.

In the next section, we describe each theoretical lens in detail, exploring their advantages and sharing relevant examples where the theory has proved useful in shedding light on various aspects of digital inequality.

## The Kaleidoscope of Theories to Study Digital Inequality

In this section, each author outlines a different theoretical approach they think may be helpful to research and understand issues related to digital inequality. The choice of the theories responds to the pertinence of the theory to study a particular dimension of the phenomenon and the expertise of the author using a particular theory or the combination of several.

### Human Development and Capability Theory (Su-Ming Khoo)

Inequality is difficult to pin down because people are different–diversity complicates our understanding of equality. Therefore, we might consider what it means to offer ‘equal access’ via digital means.

The human development (HD) paradigm and the related capability approach (CA) are interested in people’s entitlements, treating the distribution of goods and equality of opportunities as political and moral issues. CA is concerned with different individuals having unequal power to pursue well-being within their societies as a problem of injustice. Broadly, according to Sen (2009), the purpose of understanding inequality is to advance justice by reducing manifest injustices. Digital equity is achieved when digital technologies and spaces enable (and do not obstruct or reduce existing obstructions to) equitable development of different people’s capabilities to do and be what they have reason to value as a matter of justice.

Most studies relating to the CA and technology form a subset of a more extensive literature on ICT for Development (ICT4D). This literature primarily derives from locations in the global South, e.g. Chile, as an example of a country with a ‘successful digital agenda’ (Kleine 2013). Digitalisation in these territories is seen as a technical solution to societal challenges, for example, offering inclusive, quality access to higher education (HE) in the face of financial constraints, social-economic inequalities, and exclusion. However, academic and popular discussions of digital technologies are often informed by a techno-utopian ideal, which assumes that technologies must be benevolent and progressive, sometimes obscuring the fundamental ethical and social challenges and complexities of a non-ideal world.

The HD/CA is an interdisciplinary human- or people-centred approach that is analytically detailed, systematic, and oriented towards justice. Here, we distinguish the HD/CA from analyses of firm and innovation ‘capabilities’, which do not have human capabilities or justice as their focus (e.g. Andrews et al. 2018)–the latter are not relevant here.

HD/CA is particularly interested in the condition of human diversity and questions of choice. It is a critical, ethical theory containing a critique of economism and explicitly distinguishing means from ends. It hopes to shift the referent object of development to the human person as ends, not means. HD redefines development as ‘a process of expanding the real freedoms that people enjoy’ (Sen 1999: 3). ‘Real freedoms’ mean having different capabilities to function (to be, to do) and make choices. In contrast, CA focuses on opportunities and processes which prioritise people as ‘agents, not patients, in control of their own destiny’ (Sen 1999: 11). Thus, the inaugural 1990 Human Development Report (HDR) states:

People are the real wealth of a nation. The basic objective of development is to create an enabling environment for people to enjoy long, healthy, and creative lives. This may appear to be a simple truth. But it is often forgotten in the immediate concern with the accumulation of commodities and financial wealth. (United Nations Development Programme 1990: 9)

An HD/CA analysis differs from a human capital analysis, focusing not on the development of humans for the ultimate goal of economic production but on the development of functionings and capabilities that people have reason to value. It is intellectually and theoretically holistic and ambitious, claiming to be ‘the most holistic model that exists today … a practical reflection of life itself’ (ul-Haq 2003: 21). A more modest view contained in ‘Development as Freedom’ promotes a policy focus on ‘instrumental freedoms’ which include social opportunities, economic facilities, transparency guarantees, security, and political freedoms (Sen 1999).

HD/CA is a liberal, pluralist vision of equality that recognises the fundamental diversity of human beings, yet upholds every person’s equal capability for functioning and equality of effective freedom to achieve well-being. It can be described as a normative ethical perspective which offers a detailed approach to the states and activities a person ‘has reason to value’, and focuses on how to measure and evaluate progress using a wider range of indicators, such as multidimensional poverty, life expectancy, friendship, work satisfaction, happiness, and self-respect. In this way, HD/CA also pays attention to ‘adaptive preferences’ and ‘conversion factors’, which deform and structure choices to the detriment of the person in question. Moreover, HD/CA has been considered an option to enable systematic assessment of technological options that help bring questions of justice into the spotlight (see Hillerbrand et al. 2021).

Collective choice and agency are an important special topic, recognising that while HD/CA is an ethically individualist approach, individuals can generally only achieve choices in dialogue and concert with others. While some focus on ‘basic capabilities’ which map onto basic thresholds of needs and human rights, Nussbaum (2011) offers a specific approach to ten ‘central capabilities’ for a ‘good’ life: life, bodily health and integrity, senses, imagination and thought, emotions, practical reason, affiliation, other species, play, and control over one’s environment.

#### Some Useful Examples

What relevant examples of HD/CA relate to ICT, and which types of questions have been addressed? Kleine (2013) applied the CA to ICT in a general way to develop a ‘choice framework’ for understanding ICT. Kleine’s (2013) work was done in a rural community in Chile, studying the government’s digital agenda and its uptake. The CA has been used in the past 30 years to understand poverty, especially multidimensional deprivation, but it has not been used much to evaluate technologies.

One recent example considers the digitalisation of energy networks (smart grids) and automated vehicles. This study employs both a Fraserian perspective on the three dimensions of justice: distributive, cultural-recognition, and political-representation. This theory is explained in detail in the section titled ‘Tripartite Justice’ (Hillerbrand et al. 2021: 338) and Nussbaum’s (2011) ‘central capabilities’ to evaluate the potential positive and negative impacts of energy digitalisation and automated vehicles.

Further examples focusing especially on community-based participatory projects and epistemic injustice in Africa, Europe, and Latin America are richly described in a collective volume edited by Walker and Boni (2020). Some of these participatory research examples have a digital dimension, and the CA is the underlying framework of most of the case studies presented in this collection.

### Theory of Practice (Laura Czerniewicz)

In this context, inequality connotes fairness or the same distribution and access, with likely different outcomes for different people, whereas equity connotes appropriate or proportionate fairness in access and outcomes.

Bourdieu’s framework provides a way of describing students’ practices through the key concepts of ‘field’, ‘habitus’, and ‘capital’. The field explains and defines the structures or systems within which individuals attempt to achieve their outcomes. It is ‘a structured system of social positions … the nature of which defines the situation for their occupants’ (Jenkins 2002: 85). HE is one of a series of relatively autonomous worlds or fields whose complex interactions constitute society. Like all social fields, HE is a site of struggle over resources of all kinds, as it is ‘a system of forces which exist between these positions … structured internally in terms of power relations’ (Jenkins 2002: 85). Access to forms of capital is central, as ‘positions [in the field] stand in relationships of domination, subordination or equivalence (homology) to each other by virtue of the access they afford to the goods or resources (capital) which are at stake in the field’ (Jenkins 2002: 85). These positions are relational relative to specific forms of capital. Bourdieu explains that the structure of the distribution of the different types and subtypes of capital at a given moment in time represents the immanent structure of the social world, i.e., the set of constraints, inscribed in the very reality of that world, which durably governs its functioning, determining the chances of success for practices (Bourdieu 1990: 241).

Capital presents itself in four fundamental forms: economic, social, cultural, and symbolic. Economic capital refers to assets either in the form of or convertible to cash. Social capital is about connections, social obligations, and networks, i.e. who you know (or don’t know), and the advantages or disadvantages of a person. Cultural capital occurs in three states. Embodied cultural capital refers to ‘long-lasting dispositions of the mind and body’ (Bourdieu 1990: 241), expressed commonly as skills, competencies, knowledge, and representation of self-image. Objectified cultural capital refers to physical objects as ‘cultural goods which are the trace or realisation of theories or critiques of these theories’ (Bourdieu mentions pictures, books, dictionaries, instruments, machines, etc.). Institutional cultural capital is the formal recognition of knowledge, usually in the form of educational qualifications. Finally, symbolic capital is appropriated when one of the other capitals is converted to prestige, honour, reputation, and fame–recognition, value, and status. Notably, one form of capital can be converted into another. The different forms of capital are various forms of power, but the relative importance of the other forms will vary according to the field.

Habitus is how all the different constructs come together, the dynamic and shifting relationship between particular fields and capitals. Bourdieu explains that habitus is a system of durable and transposable dispositions developed in response to determining structures. An individual’s habitus is involuntary (outside of their control) and voluntary (changeable). Habitus is about identity, being in the world, and the intersection between structure and agency. It is, therefore, clear that while individuals can exercise agency, that agency is socially constrained and is exercised within existing social conventions, rules, values, and sanctions, negotiated specifically within the rules of the fields in which they operate (Czerniewicz and Brown 2012).

Bourdieu’s impact has been wide-ranging, but certain concepts, in particular, have had significant resonance: the symbolic capital which particular forms of a language bring to their speakers while other forms do not; the symbolic power and violence through which the social norms of acceptable language are reproduced, sometimes with the complicity of the speakers who are led to conform; the habitus, which embodies (literally) the tension between individual agency and social forces and occupies a position in a field with other habitus, each defined by their difference from the others.

Bourdieu treats habitus essentially as ‘a set of dispositions which incline agents to act and react in certain ways’ (Thompson 1991: 12), dispositions that sediment within us through social interaction from childhood onward, and that becomes a physical part of our nervous system. These dispositions are inculcated into us from early childhood, and they generate regular practices without being governed by any ‘rule’. The habitus is inhabited by an active human agent who is defined by the system but, crucially, is not merely its passive object. The agent engages in exchanges of symbolic power with other agents, each of whose habitus is linked to the rest in the shared field.

When Bourdieu deals with symbolic capital and power, his touchstone is often Max Weber, who described himself as a ‘political economist’. Since the political is about power, and the economic is about capital, the reference is appropriate. Bourdieu makes it clear that individuals can, in a wilful, active way, undo any identities into which they were socialised, where ‘identities’ are understood not as objective categories but as categories through which we are perceived by others with whom we come in contact, and in many cases, through which we perceive ourselves. These perceptions then affect how we are placed relative to others within a social hierarchy, or rather a network of social hierarchies. The word hierarchy itself implies an unequal standing, and it is into this standing that perceptions of us feed. Within a social field, the hierarchical positioning is determined by and determines what each of us possesses relative to others in terms of powers, goods, and rights–a combination of economic and symbolic goods. This constitutes ‘capital’ because possessing it automatically gives one the means of increasing it.

The dispositions constituting the cultivated habitus are only formed, only function, and are only valid in a field, in the relationship with a field which, as Gaston Bachelard says of the physical field, is itself a ‘field of possible forces’, a ‘dynamic situation’, in which forces are only manifested in their relationship with certain dispositions. This is why the same practices may receive opposite meanings and values in different fields, in various configurations or opposing sectors of the same field (Bachelard in Bourdieu 1990: 60).

For Bourdieu, habitus is a model for understanding how we act as agents, making deliberate choices within the parameters of a social field that accords a value to our acts, a value of which we develop an instinctive, corporeal cognition through sedimented experience (Joseph 2020).

#### Some Useful Examples

Bourdieu’s framework is perhaps the most widely used to describe and analyse inequality and digital inequality, owing to its reasonable accessibility. Some scholars prefer the term resources rather than capitals because of the association with human capital theory. Theorisations of capitals based on Bourdieu’s original framework have been accused of using a kitchen-sink approach, creating, and defining new capitals for every new interest of researchers. Similarly, scholars have debated whether digital capital is distinct from other forms of capital in the digital inequalities field or whether we should map digital onto traditional capitals (Ragnedda and Muschert 2013). Still, others contend it is not a primary capital but a secondary form of capital, similar to objects or status (Villanueva-Mansilla et al. 2015).

High-quality access is, therefore, not a separate digital capital but a secondary capital that individuals have primarily because of their economic capital (e.g. wealth). However, it can also be an outcome of cultural capital if aspects of their upbringing have socialised them into perceiving technologies to be significant (Helsper 2021). Czerniewicz and Brown (2012, 2013, 2014) offer valuable examples where Bourdieu’s theory has been used to analyse digital inequality.

### Cultural-Historical Activity Theory (CHAT) (Warren Lilley)

In this section, the potentials of cultural-historical activity theory (CHAT) are described to provoke novel insights into digital inequality within education and how this theory and its research methodologies can be harnessed towards realising more significant equity in how educators and students utilise digital technologies for teaching and learning.

The need for new approaches to addressing digital inequality is grounded in how much conventional insight overly simplifies digital inequality purely as an ‘access’ issue. These over-simplified framings often neglect the broader historical, social, and cultural aspects that perpetuate and influence how people utilise digital technologies in their diverse settings. By advocating the ‘solution’ as purely ‘access’ to more ICTs, there is a risk of further reproducing inequalities rather than finding ways to promote equity. Given this over-simplification, I discuss how CHAT can provide more nuanced insights into digital inequality as a multidimensional phenomenon in educational research.

As a concept, digital inequality is often seen as synonymous with the ‘digital divide’ (Mubarak et al. 2020; Robinson et al. 2020). This view of digital inequality is depicted as a ‘divide’ between those that have ‘access’ to use the latest ICTs (the ‘haves’) and those who do not have the same ‘access’ (the ‘have-nots’). However, this ‘access’ framing has been increasingly found wanting. For example, Mervyn et al. (2014) comparison of two UK government mobile-technology initiatives designed to aid socially excluded citizens’ access to governmental services demonstrated that merely providing mobile access did not benefit these communities to use these services. Instead, their study illustrated that by these initiatives not considering these citizens’ social and cultural contexts as well as diverse literacy needs, these mobile interventions amplified social exclusion rather than mitigated it. Similarly, Hardaker et al.’s (2017) and Tsuria’s (2020) research demonstrated how religious and gender norms could restrict women’s ability to harness digital technologies despite their ability to access them.

These studies and a plethora of others (see Robinson et al. 2020) demonstrate that the unequal experience of digital technologies is more than mere differential ‘access’ to digital resources. Instead, these studies indicate that digital inequality is a socio-cultural phenomenon wherein an individual’s potential capacity to utilise ICTs is tied to broader social structures, cultural norms, and beliefs. One theoretical approach that can account for this is CHAT, which stresses how an individual’s intentional uptake of cultural tools is both afforded and constrained by socio-cultural structures and relationships (Engeström 1987).

Premised on Vygotsky’s and Cole (1978) dialectical account of human development, CHAT emphasises the interdependent relationship between the individual and their wider community. To illustrate this, consider a formal learning environment with a teacher and students. Both have entered into this interaction to realise a socially derived motive. For the teacher, this could be financial compensation; for the student, this could be social mobility (amongst others). The key to this exchange is both parties’ reciprocal interaction is premised on the other’s participation to realise their aims: to learn, the student requires the teacher; to teach, the teacher needs the student.

Moreover, the agency of the teacher and the student to pursue these motives is afforded and constrained by various broader social and cultural factors beyond their control. For example, the teacher’s agency to employ any digital resource to instruct the students depends on broader schooling infrastructure, school board policies, or appropriacy to mandated curricula. Similarly, the students’ ability to direct the lesson or employ their digital device use is equally constrained by similar broader socio-cultural aspects, which, while not physically present, still constrain and afford their available actions within the exchange with a teacher.

Central to CHAT is that the socially derived motives for the teacher and learner and the broader socio-cultural aspects that enable or limit their actions have historically developed over time (Engeström 1987). For example, consider how classroom learning has evolved through the available tools, configurations, curricula, and learning goals–these have never stayed the same but have historically evolved to meet, realise, and challenge greater collective social motives (Säljö 2010). By underscoring both the historical and cultural aspects of human activity with semiotic and physical tools, CHAT promotes a nuanced understanding of the powerful ways in which access to digital technologies promotes unequal relationships and how these tools may further perpetuate historical and social inequalities in their use and uptake.

#### Some Useful Examples

In this regard, a notable CHAT study can be seen in Mnyanda and Mbelani’s (2018) CHAT-informed analysis of critical literacy of Grade 9 learners in Eastern Cape township schools. In mapping teachers’ and learners’ activity towards developing critical literacy, the study demonstrated that unequal proficiencies in digital media made it difficult for teachers to develop learners’ critical literacy in the classroom effectively. The study showcases how differential access, formal acknowledgement, and development of critical digital literacy skills for in-service and pre-service teachers may negatively impact critical literacy instruction within South African classrooms, especially in marginalised communities.

These insights become even more pronounced in Isaacs’ (2020) CHAT analysis of South African (RSA) digital education policy. Their analysis highlights an evolving tension in the activity of RSA educational policy development which overemphasises market-driven, performative discourses necessitating digital infrastructure for administration over socially driven discourses aimed at transforming teaching and learning of marginalised communities. For example, while policies make explicit provisions for digital technologies in education administration, they undermine their use by teachers for meaningful teaching and learning activities. The analysis concludes by suggesting that should this tension in policy development remain unresolved and current market-driven understandings pursued, further exacerbation of experienced digital and social inequalities in RSA education will continue.

Another notable study of CHAT’s ability to unearth digital inequalities can be seen in Mervyn et al. (2014) comparison of two UK government mobile-technology initiatives. By mapping the activity of these two social interventions, the researchers could showcase how these top-down approaches could not fully account for the diverse social-economic barriers and literacy needs of the marginalised communities they were aimed at. Furthermore, their analysis highlights how interventions centrally premised on the ‘neutral’ introduction of digital tools to overcome the ‘digital divide’ will likely always fall short of meeting the unique contextual inequalities designed to overcome.

This brief mention of contemporary studies illustrates that, as a research approach, CHAT can identify spaces where digital inequalities may be present when digital tools are introduced into human activities. However, what is less well-known about CHAT theory is that its dialectical understanding of development also captures a formative-research intervention methodology. As is often cited in contemporary digital intervention literature, top-down interventions fail to account for participants’ life-world complexity as participants are usually not included in these interventions’ designs or outcomes (Engeström 2011). To that end, CHAT’s research methodology of Change Laboratories (CLs) emphasises participants’ agency to direct the development and trajectory of the intervention in line with the unique demands of their context. In other words, these interventions aim to empower participants to find pathways to re-develop their activity in response to their social needs.

Several studies in digital education have found that the use of this formative-intervention research methodology was able to create more democratic, emancipatory practices with digital technologies which responded to the broader inequalities participants experienced (seeAagaard and Lund 2019; Juujärvi et al. 2016; Lund et al. 2019; Lund and Rasmussen 2008; Rasmussen and Ludvigsen 2009). If digital inequality research is premised on finding how individuals are disenfranchised from the benefits of digital technologies (Robinson et al. 2020), then digital equity research should be premised on finding socially responsive ways individuals can meaningfully benefit from their inclusion. To that end, I believe CHAT can facilitate research in both directions, which can genuinely help realise research towards a more equitable use of digital technologies.

### Affective Inequality and Affective Injustice (Aisling Crean)

In the context of education, Kotzee (2017) explores varieties of epistemic injustice (injustices related to knowledge) that crop up in the classroom, while Bacevic (2021) considers the implications of a similar phenomenon that she labels ‘epistemic positioning’ for the sociology of knowledge in a higher education culture where the participation of women and ethnic minorities is low. However, our learning processes are not purely epistemic (Boud et al. 1985); they are charged with emotions like confusion, boredom, wonder, frustration, anxiety, curiosity, and love. Therefore, this section explains how our emotional lives when learning can be *loci* of social inequalities bound up with a family of injustices known as *affective injustices* (Srinivasan 2018), or injustices bound up with unfair attitudes to the emotional lives of others. It then explores the way algorithmic decision-making used by digital technologies in online learning spaces is implicated in generating *algorithmic* affective inequalities and injustices in digital education. In a nutshell, *algorithmic inequalities* result in affective inequalities and injustices in the context of digital education and these damage the process of learning.

In contemporary philosophy, the idea that our social and emotional lives might be *loci* of a distinctive family of injustices that Srinivasan (2018) calls *affective injustice* has been explored by Whitney (2018), Archer and Mills (2019), and Archer and Matheson (2020). Srinivasan notes that in day-to-day life, we can and do consider whether emotional responses, such as anger, are apt responses to how things are or whether our anger is ‘a fitting response to how things are’ (Srinivasan 2018: 6). She argues that emotions like anger are appropriate when (a) properly motivated by a personal reason to feel anger and (b) are a proportional response to a genuine moral violation, as opposed to being a violation of someone’s wishes or desires that are not grounded in any moral values. But even when anger meets these criteria, it can be policed and silenced, discouraged, or ignored.

For example, victims of oppression are often advised to ‘let go’ of their anger or told straight out (usually by those in power) that it is ‘inappropriate’, ‘uncivil’, or simply ‘unwise’. According to Srinivasan (2018), these kinds of responses to appropriate anger constitute what she calls *affective injustice*. Whitney (2018) identifies three types of injustice that involve the lack of uptake given to the emotions of oppressed groups: *affective marginalisation*, *affective exploitation*, and *affective violence*. Archer and Mills (2019) draw on research on emotion regulation to further elucidate the nature of *affective injustice*, illustrating the kind of work imposed upon people experiencing *affective injustice* and explain why it is harmful.

This work in philosophy is in tune with work by Lynch and McLaughlin (1995) in sociology that explores debates around the nature of work, especially two interrelated kinds of work: caring- and love-labour. Finally, Archer and Matheson (2020) discuss emotional imperialism, a kind of *affective injustice* involving a dominant group imposing its culture’s emotional norms and standards on a less powerful or oppressed group. The following section discusses examples of more sophisticated digital *affective* inequalities imposed on learners by data-intensive technologies and algorithmic decision-making in the context of digital education, and frames these inequalities in terms of *affective injustice* to conceptualise and elucidate the extent of their negative impact on learning.

#### Some Useful Examples

In the context of digital education, algorithmically driven facial recognition systems are increasingly being used for securing young people’s safety, attendance monitoring, proctoring, and authenticating online learners to control access to educational content, as well as being used as indicators of student engagement and support for pedagogical practices putatively connected to concerns about well-being (Andrejevic and Selwyn 2020: 118–119). Such algorithmically driven systems are not just abstract computational processes; they also have the power to enact material realities by shaping social life to various degrees (Beer 2013; Kitchin and Dodge 2011). Beer (2017) reflects on the role of such algorithms in shaping how people are treated and judged and how, as a result, they affect outcomes and opportunities for people, while Bucher (2017) explores ‘how algorithms make people feel’, elaborating on the details of people’s personal stories of algorithms and their effects on their lived experiences, their friendships and memories, and their sense of self.

In 2020, the proctoring company ExamSoft told Black students taking exams in the USA that its software could not identify them due to ‘poor lighting’ (Chin 2021). In fact, there were usually no problems with lighting and the problem was not replicated for White students working in similar conditions; rather, racial bias working against Black skin tones was baked into the algorithm. Characterising this situation with ExamSoft as one of mere algorithmic bias against Black students underplays the character and significance of the inequality of treatment for the learning of Black students in contrast with that of White students since it ignores the affective injustice of the attendant emotional fallout for Black students and its consequences for their learning and sense of belonging. Prospective law students using proctoring software have described the emotional fallout of racially based inequality treatment by algorithms while attempting mock bar exams in stark terms (Harwood 2021). One student described how emotionally stressful the difficulties she faced were, how they interfered with her ability to perform, and how, ultimately, they left her questioning whether the law profession was for her and wondering whether it would recognise her as a person when she entered it.

If we understand such algorithms as, effectively, being optimised for Whiteness, we can see that the specific *kind* of algorithmic injustice these algorithms inflict is of a piece with the affective injustice that Whitney (2018) and Srinivasan (2018) describe [in this particular instance, Whitney (2018) characterises it as *affective marginalisation*] but is, in contrast, the result of algorithmic, rather than human, decision-making. In being optimised for Whiteness, proctoring algorithms de-prioritise and marginalise Blackness, resulting in what Whitney (2018: 495) calls ‘disabl[ed] affective sense-making in marginalised persons’. This causes significant damage to a learner’s socio-emotional learning processes, often leaving them feeling like they do not belong.

### Resources Appropriation Theory (Swati Bute)

Digital inequality can be understood as the unequal or differentiated use of the available technology, infrastructure, services, facilities, and information. It prominently exists socially, economically, educationally, culturally, and in geographically diverse societies. Digital equality, instead, is a deliberate and dedicated effort to provide digital technology, infrastructure, services, facilities, and information at a minimum cost to all citizens so that they can be informed and participate in the growth and development of their society. In such a society, the distribution and availability of digital technology, infrastructure, and services are insured without any military, geographical, or economic agenda.

Accessing technology is one thing but understanding and using that technology is another. The ability to use technology depends on the structure and setup of the society, as how and for what purpose they use technology are central aspects of understanding and achieving digital equality. What impact the technology makes on society and people is a different aspect of achieving digital equality. Therefore, digital equality is not a linear process but is multifaceted in its nature.

Jan van Dijk (2006) developed his resources and appropriation theory to better understand the concept of the digital divide, inextricably linked to digital inequality. Research within the theory can be categorised into two distinct phases: the first concerns physical access to technologies, which characterised early research (van Dijk 2006). However, as digitisation increased, the concept of access needed to move beyond the mere appropriation of digital resources and to take account of the inequalities experienced as these technologies entered people’s daily life–a concept coined the *second level divide* (van Dijk 2017). This ‘deepening divide’ emphasises that digital inequality does not end after physical access has been attained. Instead, digital inequality is further exacerbated by how individuals and communities incorporate technology shaped by different sociological dimensions such as gender, age, education, and ethnicity (Ragenedda and Muschert 2013).

The theory proposes that four main factors contribute to the quality of digital access: the dimension of motivation, physical access, cultivation of skills, and usage typologies–personal categories (e.g. age, sex, gender, ethnicity), positional categories (e.g. labour, education, household), and resources. Van Dijk (2017) distinguishes four types of access:

- Lack of any digital experience caused by lack of interest, computer fear, and indifference to new technology
- No possession of computers and network connection (material access)
- Lack of digital skills caused by insufficient user friendliness and inadequate education or social support (skill access)
- Lack of significant usage opportunities (usage access) (van Dijk 2017).

#### Some Useful Examples

A small 2020–2021 empirical study (not yet published) conducted by Swati Bute at Jagran Lakecity University in Bhopal, India, involving undergraduate and postgraduate students of journalism and communication will serve as an example. The findings are based on daily online interactions with students (e.g. participation in online classes, observation of assignment submission, and students’ exam performance). The study’s results shed light on a few critical points. At the first level of the digital divide, the participants struggled with multiple factors in accessing the infrastructure and services required to attend and participate in the online class. For example, many students could not attend classes, submit assignments, and appear in online exams because of electricity supply, not having digital devices, and not having a stable Internet connection. Some of the students were from rural areas, so they faced many infrastructure-related problems and availability of essential services-related problems.

At the second level of the digital divide, students’ behavioural and contextual issues were responsible for the lack of interest in online classes. Not attending online classes regularly had sometimes to do with household atmosphere and household work; not participating in the online classes, e.g. keeping camera and audio off during the classes, was due amongst other things to household issues as well as economic constraints; giving wrong reasons for late submission of assignments and online exam papers; saving mobile data for other personal online activities; and remaining active on social media platforms were some of the behaviours observed. In addition, during the first phase of the pandemic, due to uncertainty, students were shocked, fearful, and traumatised. Many students were infected by the virus during the second phase of the pandemic; and some students had lost family members, no wonder that students remained silent and invisible.

### Critical Pedagogy and Digital Liberation: a Freirean Approach (Sandra Abegglen, Tom Burns, and Sandra Sinfield)

The [online] classroom remains the most radical [online] space of possibility in the academy. (hooks 1994: 12)

In this section, we continue the discussion on understanding digital inequality through Paulo Freire’s lens and his idea of critical pedagogy. We are building on the premises presented in the ‘Critical Realism’ and the ‘Human Development and Capability Theory’ sections that put forward that digital inequality is multifaceted and part of larger social inequalities, positing that it is useful to think about what it means to have ‘equal access’, not just in terms of access to technology and broadband, but access to the academic and cultural capital that allows educators and students to use the digital for research, study, voice, and liberation. We ask what it means to be an academic and student in a world that relies on digital technology, problematising the notion that technology automatically grants access and enhances teaching and learning (Bayne 2015), suggesting instead that both educators and students need to learn how to harness digital education for liberatory purposes (Freire 2018; Stommel 2014)–for agency and ‘action’.

As outlined in the ‘Introduction’ section of this article, education takes place in a supercomplex world (Barnett 2000a; Abegglen et al. 2020a, b). Thus, as Barnett (2000a, b) asserts, the main pedagogical task of a university is not to transmit knowledge but to develop students’ attributes appropriate to the conditions of supercomplexity. In a later paper, Barnett (2004) calls for a pedagogy that prepares learners for an unknown future, a pedagogy that fosters and supports human qualities that help students in standing up to the world and engaging with it purposefully. ‘What is called for, therefore, is a creative knowing in situ.’ (Barnett 2004: 251). Concerning digital education, then, we need to problematize the way that we discuss digital inclusion and equality–to help us rethink learning and teaching itself in more equal terms.

Paulo Freire, the Brazilian educator and philosopher, was a fierce advocate of critical pedagogy, a philosophy of education and a social movement that developed and applied concepts from critical theory and related traditions to the field of education and the study of culture, proposing a more equal relationship between teacher, student, and society. While most of Freire’s work, including *Pedagogy of the Oppressed* (2018), was written before digital technology and the Internet entered the classroom, the writing offers valuable pointers for rethinking digital inequalities in education (see, for example, Johnston et al. 2021).

Freire (1972/2018) posits that education, as with technology, is not neutral, objective, measurable, and apolitical. Those who are oppressed need to be given the freedom to express themselves, in their own words, in their own spaces. There is something profound in Freire’s attempt to help the oppressed fight back to regain their power–to find their words–to have their humanity recognised. If we apply this to digital inequalities, we can conceive of an approach that does not construct an idealised model of a ‘technology-tooled-up’ student, a digital native, able to afford and navigate the World Wide Web seamlessly, but rather an approach that acknowledges that that particular idealised model is itself not ‘neutral’ but serves to dehumanise further and disempower those on the other side of the digital divide. Only when that shift has been made can we start creating digital equity.

If we are to truly create equal access to the digital and the digital world, we perhaps should start as Freire started, with the actual students in all their humanity, their strengths, their frailties, and their burning hopes and fears (Farag et al. 2021). If we allow lecturers to set challenges and tasks that enable students to play with and experiment with the digital for self-expression, exploration, and creative emergence, then students learn to use the tools they have for themselves–they start to become digital more on their terms.

If we apply this perspective – together with a liberatory thrust – to digital inequality, rather than viewing the students only in terms of what they are not: not traditional, not prepared for higher education, not in a position of privilege or advantage’ (Smit 2012: 370) – and not digital natives – we can tackle digital equality more positively. We bridge the digital divide not by ‘remediating’ students’ lack of digital proficiency but with a ‘minimally invasive education’ (Mitra and Rana 2001) process akin to Sugatra Mitra and his Hole in the Wall (Mitra 2012) experiment.

#### Some Useful Examples

In 1999, Mitra and his team at NIIT University, Kalkaji, New Delhi, India, literally carved a ‘Hole in the Wall’ that separated the university from the slum next door (Mitra 2012). Through the hole, slum children had free access to a computer. With no prior experience but driven by their curiosity and the freedom to explore, students learned to use the computer, surf the web, and develop knowledge and skills–without the intervention of a teacher. If we apply Mitra’s philosophy to our students and their agency concerning developing digital literacy more on their terms, yes, we need to provide access to computing equipment, but more importantly, we need to accept the students as capable of driving their own learning, without the need for an all-knowing lecturer. This leads us to discuss our own ‘Develop a Digital Me’ project (Burns et al. 2018).

In our undergraduate teaching, we challenged our students with developing a ‘Digital Me’ (Burns et al. 2018). Rather than quizzing students about their digital knowledge and skills, we asked them to use an unfamiliar digital tool to make a digital artefact that would introduce them to the other students in the group. This task was deliberately evasive–students could introduce themselves digitally, or they could introduce their digital selves, or some combination of the two. We built class time in the computing labs, supplied some senior students as mentors, and asked the students to be creative and have fun. Near Christmas, rather than an assessment point, we had a celebratory ‘party’ that incorporated an exhibition of their digital artefacts. The students enjoyed showcasing their work–they entertained and supported each other–and they delighted and surprised us. Most importantly, however, they engaged themselves in authentic digital education–as a liberatory endeavour.

We are situated within and confronted by an education system that labels our students–and often ourselves–as ‘deficit’ and in need of ‘training’, especially in digital literacies. We argue that the twenty-first-century educators need to make the space and place in the curriculum for creative opportunities for emergent learning to counter current educational narratives–especially with respect to who is included seamlessly in academia and who is systematically ‘othered’ and excluded. As Sugata Mitra (2012) has demonstrated, and as we argue, we cross the digital divide by believing in learners–and recognising them as the creative human beings they are.

Why does this matter? It matters because the supercomplexity of the world and of education (Abegglen et al, 2020a) is more challenging to those marginalised and on the periphery. It matters to those students who come from ‘non-advantaged’ backgrounds; they, and their parents, are more prone to zero-hour contracts and minimum wages and more marginalised even than in traditional manual labour jobs. It matters that when talking about students, we cannot refer to them as a single, homogenous, unified group. Instead, we need to acknowledge that there is an element of uncertainty and fragility and thus strangeness about our unequal students (Lillis 2001).

This demands imagination, creativity, openness, and ingenuity on the part of the staff on the ground and of institutions themselves. We need structures and processes that facilitate the student’s ability to participate and make their accommodations with discourses of power and exclusion. Thus, we need a more significant ontological shift in pedagogy–and digital pedagogy and access. We need a practice that is supportive of difference and allows us to holistically include students–all students–so that they can participate with agency while successfully holding on to their subjectivity in the supercomplex reality we all live in.

### Tripartite Justice (Caroline Kuhn)

This approach to social justice, envisioned by Nancy Fraser (2008a, b), aims to achieve participation parity encouraging a multidimensional perspective to addressing social injustice. Distinguishing different kinds of injustices–economic, cultural, and political–is critical because they need to be challenged through different kinds of tactics. In Fraser’s view, the threat of injustice is to the ability of people to participate as a legitimate member of society, at equal footing with others. She defines participatory justice in education as:

Social arrangements that permit all to participate as peers in social life. On the view of justice as participatory parity, overcoming injustice means dismantling institutionalised obstacles that prevent some people from participating on a par with others, as full partners in social interaction. (Fraser 2007: 27)

Economic injustices are derived from the economic structure; thus, they require a politics of redistribution. They involve exploitation and economic marginalisation, e.g. being confined to work that is undesirable and poorly paid, or not having work and being deprived of an adequate living standard. Therefore, people are indirectly being denied from having meaningful connectivity (a measure of whether someone can regularly access the Internet on an appropriate device with sufficient data and a fast connection) (Namakula and Nsekanabo 2020).^1^ Cultural injustices prevent people from interacting in terms of parity by institutionalised hierarchies of cultural value related to cultural domination and non-recognition, that is, being invisible by hegemonic representational, communicative, and interpretative practices (Power 2012). They can be addressed through a politics of recognition.

Lastly, political injustices are a consequence of the former two injustices because people who are mis-recognised and endure material maldistribution are unlikely to engage in civic and political activities. Misdiagnosis of an injustice can result in strategies that may not only be ineffectual but also have the potential to produce additional injustices. Fraser (2008a, b) argues that these forms of injustices rarely exist in their pure form but separating them provides heuristic advantages to understand, for example, the match or mismatch between inequalities and existing strategies to address them (Power 2012).

By using Fraser’s (2008a, b) theory of participatory parity, we can attend to different kinds of injustices, and it is possible to craft a normative decentred framework that does not privilege technology but structural injustices that are connected to larger systems of institutionalised oppressions. The aim of using this theory in a datafied society, for example, is to decentralise big data and data-driven technologies from the debate on discrimination and recognise the broader forms of systemic oppression and injustices that produce both mediated and unmediated forms of discrimination.

Fraser (2008a, b) considers that injustices are historically contingent ideas, and it is critical, therefore, to not only focus on what is unequal but more so on ‘who’ is unequal/unjust and ‘how’ inequality is imbued in political institutions. This entails a relational understanding of justice. With this broader understanding, it will be possible to offer a normative model that is decentred from the technology and thus, permits a broader understanding of injustices supporting individuals to participate on equal footing in social life. Fraser (2008a: 405) argues that ‘overcoming injustice means dismantling institutionalised obstacles that prevent some people from participating on par with others, as full partners in social interaction’.

This model can be useful for understanding technology-mediated discrimination (Peña and Niklas 2019), e.g. algorithmic discrimination that is not centred on the technology per se but on the power relations that define the value structure of society at large. Thus, discrimination is not seen only through technology even though technical discrimination matters. Discrimination mediated by technology exists alongside other forms of discrimination that contribute to the systemic marginalisation of individuals and groups marked by social differences. Problems related to data-driven technologies, thus, data justice, are examples of such multifaceted issues.

#### Some Useful Examples

Data-driven technologies can be conceptualised as one amongst many discrimination mechanisms, and data-driven discrimination is one facet of an unjust society. Peña and Niklas (2019) questioned in their study to what extent does a decentred (of the technology) discourse exist in the real world? Their research explored how European civil society understands data-driven discrimination and connects between data, discrimination, and inequalities. For that, they analysed ‘the terrain and texture of civil society discourse on data and data-driven technologies, including when and how technology plays a role in civil society organisation’s work on discrimination as well as who is impacted and how discrimination can be prevented’ (Peña and Niklas 2019: 887). To answer the question of how European civil society understands and encounters automated computer systems, data, and discrimination, and to what extent maldistribution, misrecognition, or/and misrepresentation factors play a role on these understandings or encounters, they use Fraser’s (2008a) tripartite social justice model.

Focusing on the technology seems to prioritise technical forms of discrimination or unfairness at the expense of other non-digital discriminatory techniques faced by individuals or groups who systematically bear the risks and harms of a discriminatory society. So, while techniques may vary over time, discrimination’s target may stay unchanged. What remains constant is the marginality and deprivation experienced by socially silenced groups. ‘Who’ matters as much as ‘how’ as Fraser (2008a) insists. In other words, unmediated discrimination exists alongside technologically mediated techniques of discrimination. Algorithmic discrimination and exclusionary automated systems represent one element of a larger ecosystem of discriminatory practices and procedures, and any diagnosis of problems or prescription for remedies would benefit from some measure of reflexivity concerning this ecosystem (Peña and Niklas 2019: 887).

Another example is explained in ‘Participatory Parity and Emerging Technologies’ (Bozalek 2017).

Students who make use of their own devices may find themselves excluded by the banning of mobile devices such as mobile phones in lecture theatres, for example. Or those who do not have access to Internet services in their homes may find themselves being excluded from courses, which are blended or offered fully online. (Bozalek 2017: 92)

Following Fraser (2003), injustices can be addressed through affirmative or transformative social arrangement; the former facilitates the outcomes and the latter addresses the structural causes of the injustice. From an affirmative perspective, socially just pedagogies would be achieved by addressing education inequitable outcomes by making ameliorative changes to how teaching and learning are practised. In other words, the changes that the socially just pedagogies would affect would not disturb the underlying structures that generate social inequities but would address what Fraser (2003: 74) refers to as the ‘end-state outcomes’.

On the other hand, transformative approaches to socially just pedagogies would involve practices which address the root causes of maldistribution, misrecognition, and misrepresentation in the three dimensions. Examples of affirmative arrangements can be the case of the lecturer that brings their device to solve the problem of lack of technology. Whereas some other teachers made rather transformative arrangements, such as ensuring that the institution provided adequate Internet access to all students in his/her/their class and to all devices that they brought themselves, by insisting that the institution install a wireless router in the classroom (Fraser 2003: 98).

### Critical Realism and Realist Social Theory (Caroline Kuhn)

An essential aspect of digital inequality is studying and intervening in the organisation of social structures embedded in digital technology infrastructures. However, this is not a straightforward endeavour for many reasons, one of which is the invisibility of social structures, power relations, and the prominent presence of ‘commonsensical’ ideas, so commonly deployed in the world of technology use. Critical realism (CR) is a philosophical approach to understanding (social) science initially developed by Roy Bhaskar (1998, 2008). In contrast to positivism’s methodological foundation and poststructuralism’s epistemological foundation, CR argues that (social) science should be built from an explicit ontology. In this respect, critical realists start any investigation by acknowledging that the world exists independently of the knower. Our knowledge of the world is historical, partial, and fallible. Knowledge only describes the world partially and at a particular moment in time. Therefore, any critical realist research starts from the premise that social reality is much more than what catches the eye and what the researcher can observe and grasp from the empirical data collected.

An illustrative example of the transitive nature of any knowledge that seeks to understand the world is the shift from a geocentric model of the universe to a heliocentric one. Before Copernicus, the system of the universe was explained using a geocentric model. Still, given the creation of more powerful telescopes and the availability of systematic data from former astronomers, Copernicus discovered and created a model of the Universe that revolutionised science and was later refined by Galileo. This story is more complex than this, but the point I want to make is that our knowledge is historical and theory/concept dependent, thus, transitive, unlike the existence of the world, which does not depend on any theory or knowledge that seeks to explain it. The world exists, waiting to be explored but never fully understood.

CR conceives social reality as stratified, emergent, constantly transformed, and/or reproduced by agents (Archer 2007). Structure, for CR, precedes agents but are consistently reproduced or transformed by agents. The world has different stratas, i.e. the empirical, the actual, and the real. An iceberg is a helpful metaphor for understanding the stratification of the world. The tip of the iceberg represents only 10% of the whole mass of the iceberg, and it is the empirical layer that the knower can observe through the senses. Underneath the water lies 90% of the rest of reality. The actual level consists of the events that occur independently of them being observed by the knower. The deepest level is the real, constituted by what critical realists call generative mechanisms or powers. These generative mechanisms interact in myriad ways to produce the events at the actual level and observed at the empirical level. These mechanisms or generative powers are the properties of social and cultural structures that emerge when individuals or groups interact with society. These mechanisms are relatively enduring and make things happen in the world, namely in the social world of our concerns.

At its core, critical realism offers a theory of being and existence (ontology), but it takes a more open position to the theory of knowledge (epistemology) used to explain social phenomena.^2^ Therefore, an array of approaches has developed that offers a theoretical framework for social research. Because they are not theories in specific disciplines nor theories relating to particular aspects of society, these approaches are generally known as ‘meta-theories’ (Archer 2013). Critical realist social theories include but are not limited to the transformational model of social activity (Bhaskar 2016); the morphogenetic approach to the interplay of structure, culture, and agency (Archer 1995, 2013); critical discourse analysis (Chouliaraki et al. 1999); critical realist feminism (Van Ingen et al. 2020); and critical realist Marxism (Brown et al. 2002). In short, under a CR framework, the researcher has an array of social theories to choose from. However, the choice is contextual, historical, and contingent.

For example, in the context of online learning during the pandemic, different barriers must be addressed if online learning were to become an equaliser. It is not only about accessing online learning through a device but also providing socio-cultural and economic support to overcome different constraints emerging from the context. It is known that students’ social-economic status and access to learning are connected to structural issues in society: poverty, social disadvantage, gender, and race, amongst others. To address equity in access to education and its transformative capacity, it is vital to uncover these structural aspects that constrain learners’ needs. CR and the chosen theory can aid in this process. This will allow us to design strategies that can be put in place to tackle these structural issues of inequity.

#### Some Useful Examples

To understand students’ lack of reflexive engagement with open and participatory tools in an academic setting (HE), Kuhn (2022) used a kaleidoscopic approach. The instrument was built with a number of mirrors to shed light on ‘why’ and ‘how’ questions. The kaleidoscope combined a CR understanding of the world together with realist social theory (Archer 1995) to explore students’ interaction with the technologies at the institution. In addition, it integrated the transformational model of technical activity (Lawson 2007) and the capability approach (Sen 1999; Nussbaum 2000) to come up with two causal pathways that explained which are the possible mechanisms responsible for students’ lack of reflexive digital engagement.

**Fig. 1:**
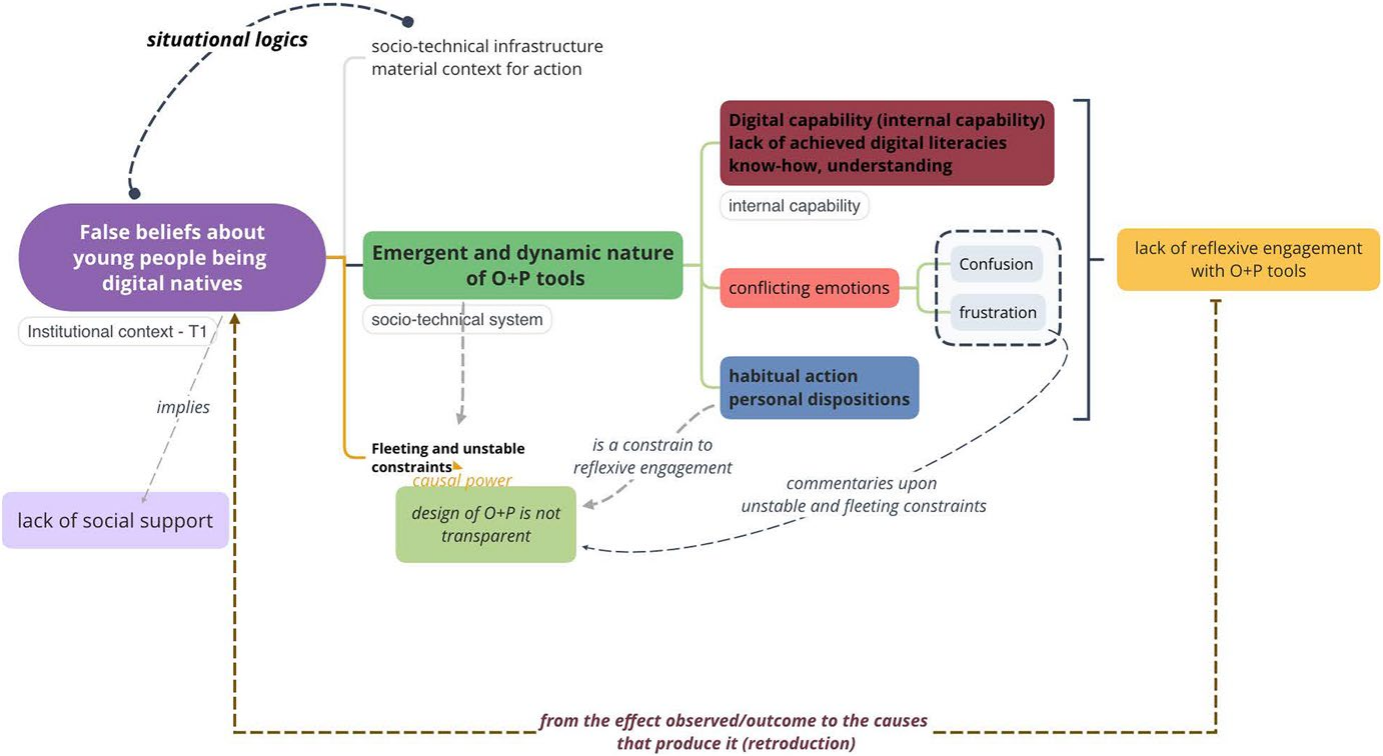
Causal pathways of students’ lack of engagement with open and participatory tools in HE (Kuhn 2022)

Figure 1 presents one of the causal pathways that illustrates the combination of a number of mechanisms; amongst them is the culture of the institution (lecturers’ false beliefs about young people being digital natives and the culture of assessment prevalent in the School where the study was made). It also shows how the sociality of open and participatory tools within the institution (the position of these tools in the institution's context of use), the valued goals of students and their conflicting emotions towards novel digital practices, interact. These interactions produce as an outcome a lack of reflexive engagement with open and participatory tools.

The focus of the study was not the tools per se but the social and cultural conditions with which students interacted that led to the outcome observed, i.e. a lack of reflexive engagement with open and participatory tools in the university context. The use of the capability approach served to conceptualise and point out the importance that having a valued goal has for the student, as a force to overcome different socio-technical constraints.

Another interesting example is presented by Eynon (2022), who explores the relationship between Internet use and social inequality. The study explores how people use the Internet, how people can exert agency by harnessing the affordances of the Internet, and the structural conditions that constrain or enable what people can achieve. In this study, using CR and RST, it was possible to understand the complex interplay of agency and structure to explain the outcomes of Internet use for different individuals. The advantage of such findings is that it promotes a focus beyond access and skills in digital inclusion policies.

## Discussion

A kaleidoscope can serve as an analytical tool to examine and critique the constitutive elements of digital inequalities using the intersection of different theories and finding generative connections to shed light on different dimensions of digital inequality so that we can decentre our attention from emerging technologies without dismissing them. For example, when turning the kaleidoscope, we might shed light to issues that have not much to do with the digital technology as such, but with social issues of misrepresentation and misrecognition of vulnerable individuals. This, in turn, constraints people’s access to and participation in the digital economy, e.g. in endeavours of knowledge production so germane to education.

For this, we would need to craft a kaleidoscope using three mirrors (theories), e.g. one being the tripartite justice model proposed by Fraser (2008a, b) that sheds light on the cultural (misrecognition) and political (misrepresentation) injustices. The other mirror could be the human development and capability theory to critique the consequences of being denied one of the most central capabilities, namely, being capable to make meaningful epistemic contributions to the common pool of knowledge. The other theory could be critical pedagogy to find alternative pedagogical approaches to remedy such injustices. In crafting this kaleidoscope using this particular intersection of theories, we are able to connect injustices that are not necessarily related only with the digital but which consequences affect the participation in the digital sphere. Thus, the kaleidoscope aids researchers to explore and understand the tensions between the social, the political, and the technological.

It becomes evident that such a complex situation like the one described above epitomises a critical attitude whereby the researcher inquiries into the digital world but is not only concerned with the digital. Instead, they scrutinise how more pervasive and elusive power relations are partially responsible for the misrepresentation and misrecognition of those who might choose or are forced to dwell on the periphery, as well as how that situation excludes them from participating in the digital knowledge economy. It is now well understood that digital inequality and exclusion cannot be analysed apart from the offline circumstances of individuals and groups. Thus, the specific forms of digital exclusion map onto particular kinds of offline disadvantage. This is what the postdigital stresses: digital/analogue and human/machine cannot be separated anymore; instead, they are in constant tension and entangled, shaping one another. Therefore, being able to explore the interplay between offline/online and human/machine can be helpful to postdigital educational research.

In this article, through using collaborative writing, we have put together a toolkit for researchers who want to choose a kaleidoscopic approach to studying digital inequality. We have presented an overview of some theories and the possibility of finding generative intersections and connections to craft a powerful kaleidoscope that serves to inquire into the world of digital inequalities examining and critiquing its constitutive elements, its theoretical approaches, and its consequences for society more generally. In addition to the detailed description of the theories, we present the reader with a table that summarises the usefulness of each theory so that choosing which to use for your kaleidoscope can become clearer.

The mirrors (how many and which) you will use to craft your kaleidoscope is a matter of professional choice shaped by the nature of the problem and your positionality. Taking inspiration from Ursula Franklin (1999), we have envisioned the kaleidoscope not as a prescriptive but as a holistic tool. Holistic tools, Franklin argues, are tools related to the notion of craft, where the artisan controls the process of their own work. Artisans, as researchers, make decisions on their own terms drawing on their experience and positionality. In short, the theoretical kaleidoscope is a holistic tool for the researcher to be as much in control as possible of their work. We cannot forget that anybody’s perspective will necessarily be limited by consideration of scope, feasibility, and context. A perspective by definition is a *particular* way of considering something.

We need to be reminded that digital inequalities, in postdigital times, are complex and they entail nuanced, thus hard to grasp tensions between offline and online, human and machine, and analogue and digital. Although we present a number of theories for reasons we have explained above, we do not argue that these are the only ones that are available to the researcher. There are many others to explore, and depending on the nature of the problem and the context, as well as the experience of the researcher, some intersections of theories will work better than others. You are the artisan, you are in charge of your kaleidoscope!

In Table 1, we illustrate the strengths and key aspects of the different theories that the researcher can choose to assemble their kaleidoscope.

**Table 1:** An overview of theories for understanding digital inequality

Theory	Description
Capability approach	• Accommodates diversity and plurality of needs and outcomes • Asserts ethical individualism (every person matters) while appreciating the embeddedness of each person in relationality and context • Distinguishes means and ends • Focused on evaluative judgement (are things getting better or worse?) • ‘Thick’ conception of ethics focused on human well-being and flourishing • The integration of feminism and decoloniality challenges the universalism of the HD/CA’s humanistic perspective (e.g. Khader, 2018)
Theory of practice	• Demystifies the link between hardworking people and success. Social and cultural capital is useful to shed light to injustices that are related with the context of people and how differences in education lead to other differences in life, e.g. digital inequality • Theory of habitus stresses the huge influence of education upon one's ability to attain success • Helps to show that inequality is not a naturally occurring phenomena but it is related to a lack of social/cultural/economic/symbolic capital
Cultural-historical activity theory	• Emphasises the interdependent relationship between the individual and their wider community • Sees digital inequality as a cultural-historical phenomenon • Highlights how no ‘tool’ (whether digital or physical) is ‘neutral’ • Promotes research aimed at exploring how digital inequality is experienced • Methodology of formative-research interventions aimed at equity • Intervention based on contradiction surfaced by community members, that is, not imposed and therefore likely to last longer and gain more traction • Dialectical rather than binary logic; contradictions as a site of dynamic change. View of contradictions as progressive
Critical pedagogy and digital liberation	• ​Highlights digital inequality as part of larger social inequalities • Sees technology as not neutral and not a panacea: it requires interrogation and creative approaches to utilise digital education for laboratory purposes • Allows students to drive their learning via authentic and engaging co-created digital ‘tasks’ • Is not just about access to technology but about harnessing technology for agency. If approached this way, it does not privilege traditional academic or cultural capital but increases access and inclusion • Links with bell hooks (1994) and education as a process of hope and freedom. Is aspirational and emergent–suitable for a supercomplex world–and the often supercomplex positionalities and experiences of students
Resource appropriation theory	• Talks about different levels of the digital divide. This plays an essential role in addressing the nuances of the phenomenon, and thus, it supports the researcher in making informed choices in society • Posits that the digital divide is not a static and a permanent condition but fluid. Stresses the need to study digital inequalities in different situations and societies, that is, in different settings
Tripartite justice	• Offers the possibility to study inequalities decentering the study from technology. Focuses the attention on deeper socio-cultural structures • Entails a multidimensional understanding of justice which aids the researchers in addressing the multifaceted nature of discrimination by into the social arrangements that underlie the many injustices • Allows the researcher to recognise other wrongdoings related to questions of ‘who’ perpetuates the injustice and ‘how’ different injustices occur
Critical realism and realist social theory	• Answers questions of why and how attempting to address the root cause and not so much the symptoms of the problem • Explores the interplay of culture, structure, and agency • Addresses structural issues in society
Affective inequality and affective injustice	• Stresses the affective or emotional dimensions of learning and shows how emotional dimensions of learning, like our emotional lives more generally, can be *loci* of injustice • Helps explore effective inequalities and injustices when using data-intensive technologies for digital education • Allows a more sophisticated analysis of cases where digital technologies used for proctoring are not simply biassed, but have inflicted specifically affective injustice on already marginalised people, especially people of colour

## Conclusion

Digital inequality is a pluralistic construct, multidimensional, and contextual. Each turn of the kaleidoscope allows the researcher to examine and critique the constitutive elements (online and offline which are not exclusive but intertwined). We acknowledge as do Peters and Besley (2019: 30) that the postdigital is not a chronological term but rather a critical attitude to examine ‘the ideology of digitalism’, its concomitant inequalities, and the huge losses it entails for human flourishing.

Digital inequality is nothing new, but its significance has augmented in the sector of HE during and after the pandemic. We believe, as do White (2009) and others (see Hayes 2021; Jandrić et al. 2018; Knox 2019), that when considering the digital, its pitfalls, and affordances, a robust toolkit that aids the researcher to focus on the broader socio-cultural questions regarding people’s lack of access to economic, cultural, social, and political power is needed.

We further argue, in line with Knox (2019), that there is a need to broaden the scope of educational research on socio-technical systems within which the project of digital education is constituted. This requires mobilising the intersection of different theoretical perspectives through a kaleidoscopic approach that allow us to transcend the tendency to understand technology, in particular educational technology, in terms of the newest gadget and the prevailing idea of an outdated educational system that needs to constantly catch up with the latest trend in technology. The educational system will always be running to understand the implications of the postdigital scenario in a way that remains open to different possibilities for humanity.

Instead, what is required is to explore, at a relational level, how power relations are (mal)distributed, shaping people’s experience when engaging in uneven ways with technologies. As Wakunuma (2019) upholds, despite the positives of the digitisation agenda, there are also negative aspects which have to be addressed in the form of ethical concerns. In particular, we argue in line with Wakunuma for an urgent need to explore the aspect of power in light of the digital transformation of the Global South. This, we sustain, is a critical task if we wish to shed light on the stark injustices and inequalities that are taking place now but are not new; they have only become more prevalent and urgent since the pandemic. We ought, as researchers, to transcend the idea of a tempting and seductive novelty and linear progress that seems to be implicit in the digital if our aim is to strengthen the social justice agenda in HE in the Global South or North, as both are in the midst of a severe crisis concerning digital injustices and its accompanying consequences for education.

For doing this, we have envisioned a toolkit for researchers consisting of some theories (it is not an exhaustive list), useful examples, and a summary of practical advantages of each theory. It will be the researcher, given her/his/their positionality and professional experience, that will craft the kaleidoscope in a particular way, to shed light on the problem at stake. Looking through a kaleidoscope can seem to be slippery and tricky; sometimes you do not see what you expect but even then it is a matter of keep on trying. Maybe you need to use different theories, maybe you need two instead of three, or maybe you need to change the object of analysis. The kaleidoscope is not the only tool to use in your research project; you also need to choose the right ontological and epistemological approach, the best methods to generate valuable data that you can then introduce into your kaleidoscope to see what unexpected patterns, and incredible images you can create. It is a holistic process where the kaleidoscope can serve your purpose to discover some of the tensions that have been overlooked perpetuating insidious injustices in society. We are not suggesting that with the kaleidoscope researchers will be able to understand and uncover everything. Instead, it is a tool to enable us to see more dimensions of digital inequality that have remained rather invisible and therefore difficult to understand and address.

## Open Review 1: the Glitter and Gloom of Kaleidoscope Research (Petar Jandrić)

When I received an early idea for this article from Caroline Kuhn and the co-authors, I immediately fell in love. Kaleidoscope research is a breath of fresh air in often-stale literature on research methods: highly creative, visually attractive, and above all pedagogical. Reminiscence of my own childhood play with kaleidoscope toys brought about some warm feelings. I even tried to find a new kaleidoscope for my 9 years old son Toma! While I could not find a new kaleidoscope in Zagreb’s toy shops, my Mum managed to find a couple of old, half-broken kaleidoscopes in the attic. A hipster move, perhaps, but Toma at least managed to taste a bit of history.

While we played with the toys, Toma asked: So each kaleidoscope is different, right? I wasn’t sure of the answer, so I quickly looked it up.

A kaleidoscope (/kəˈlaɪdəskoʊp/) is an optical instrument with two or more reflecting surfaces (or mirrors) tilted to each other at an angle, so that one or more (parts of) objects on one end of these mirrors are shown as a regular symmetrical pattern when viewed from the other end, due to repeated reflection. (Wikipedia 2023)

Sharp little fellow has hit the nail on the head. A kaleidoscope can provide an almost indefinite number of different images. Nevertheless, those images depend on the reflecting surfaces, angles, and objects in the kaleidoscope. However rich and varied, these images are indeed predefined by the physical setup of the kaleidoscope.

Back to work, researchers’ choice of approaches and theories in a kaleidoscope will always create a unique optic. However varied, this optic will be based on approaches and theories that we include and will not be based on those that we excluded (or, will be based by absence). The main theme of this paper, inequality, is also about inclusions and exclusions. Back to square one, the kaleidoscope approach to postdigital research does not escape the eternal dichotomy between inclusion and exclusion. However, it does reconfigure this dichotomy, and I believe that this reconfiguration is important in several ways.

First, typical (postdigital) research is based on one or two methodologies; studies that intersect three or more research approaches are few and far in between. With full recognition of issues arising from commensurability of research methods (see Jandrić 2021), the kaleidoscope pushes researchers towards more varied approaches–and that’s a good thing.

Second, the kaleidoscope offers many ways of combining chosen methodologies. Carefully avoiding the mixing of proverbial apples and oranges (another shout-out to commensurability), it still offers an inspiration to develop fresh and unusual mixes, perhaps those that we would not think of otherwise.

Third, the kaleidoscope is (at least in my aged mind) such a beautiful picture, which is itself deeply pedagogical. Displaying links and connections between approaches and methods that I never thought of, it helped me think of postdigital research in a new way. Cannot wait to test it with my students on Research Methods course!

While I could continue this praise for much longer, I do feel responsible to end with some words of caution. One is the need to beware of various inclusions and exclusions inherent in the method (see Bayne in Networked Learning Editorial Collective et al. 2021). The other is the need to resist definitions of the kaleidoscope research method (see Jandrić and Ford 2022; Jandrić 2022). Yet another is to beware of the apparent infinity of kaleidoscopic opportunity–all that glitters is not gold (Jandrić et al. 2022a, b). And yet another is to think carefully through connections between this theoretical richness and practical reality (hopefully through the concept of critical praxis) (McLaren and Jandrić 2020).

We should not get too infatuated by the glaze and glitter of kaleidoscopic research methods and we should neither be put off by their gloom(ier) sides. A proper response, especially for a reviewer, usually lies somewhere around a moderate middle. Yet I cannot help my excitement with the new, shiny metaphor that evokes such warm feelings. For better or worse, I do wish to explore it further in my future work!

## Open Review 2: David Brewster’s Kaleidoscope: Precision and ‘Supercomplexity’ (Jeremy Knox)

The assembled group of theoretical approaches in this article appears to offer much for the study of digital inequality, including the *capability approach*, which emphasises the ‘distribution of goods and equality of opportunities as political and moral issues’, to *cultural-historical activity theory*, which draws attention to ‘historical and social inequalities’. In each of the eight theoretical contributions, a succinct outline is followed by concise examples, resulting in a clear sense of the potential value to the study of digital inequality. The proposition that warrants further reflection in this paper, however, is not contained within any one of these theoretical frameworks, but in the titular suggestion of their methodological combination, through the analogy of the ‘kaleidoscope’.

It seems pertinent, as I write this open review in my office at the University of Edinburgh, to acknowledge that the kaleidoscope was invented in the early 1800s by a Scotsman, David Brewster, who was not only an alumnus of the university in which I now sit, but also later in his life, the Principal. Not far from the view outside my window is the Royal Society of Edinburgh, where a prototype of the device was first introduced. That Brewster could engage such audience was because he was a relatively prominent scientist of the time, working in the field of wave optics, in which he is credited with several discoveries, including ‘Brewster’s law’, which defined relationships between light waves. As I ponder the value of a ‘theoretical kaleidoscope’, I am conscious that some of the very first kaleidoscopic views were perhaps of the very same Edinburgh skyline that presents itself before me now.

The scientific approach underpinning the invention of the kaleidoscope (Brewster’s idol was apparently Sir Isaac Newton, but he was curiously an anti-Darwinist) suggests to me two brief reflections (no pun intended). First is the precision. As Brewster’s *Treatise on the Kaleidoscope* (1819) makes clear, the device required a meticulous positioning of three key elements: the reflectors, the object, and the eye of the viewer. Of the latter, he suggests: ‘That out of an infinite number of positions for the eye, there was *only one* where the symmetry was perfect’ (Brewster 1819: 5) (emphasis original). The complex images produced by a kaleidoscope, in other words, do not result from much in the way of ‘creative licence’ with the arrangement of its key elements. If the theories outlined in this paper are the reflectors of the kaleidoscope, as suggested, they may need to be positioned and aligned in very precise and predefined orientations in order to produce the proposed beauty of a kaleidoscopic insight. Such precision suggests a rigour in the combination of theory that may be antithetical to the study of ‘supercomplex phenomenon’.

And this leads to the second reflection: complexity. Brewster’s fastidious composition of reflectors, object, and eye seems to imply a quite rigid relationship between the three, and a rather dualistic distinction between subject and object. Furthermore, the complex image one encounters by using the device is assumed to derive, not from the object itself, but from the array of reflections produced by reflectors. In other words, the object is *made* complex, rather than being assumed to be complex itself. Brewster states:

The fundamental principle, therefore, of the Kaleidoscope is that it produces symmetrical and beautiful pictures, by converting simple into compound or beautiful forms, and arranging, by successive reflections, into one perfect whole. (Brewster 1819: 17)

For Brewster then, the kaleidoscope converts the simple into the compound, and the results are undeniably beautiful. However, interpreted thusly, the kaleidoscope does something fundamentally different from what is proposed in this paper, which is to assume ‘supercomplexity’ as intrinsic to the object itself (in this case digital inequality), and to draw on multiple theoretical frameworks to discern its intricacy, nuance, and convolution. The cautionary tale for the mixing of theories here would then be one in which the mixture itself becomes the focus of complexity, difficulty, and attention, rather than the object of study itself.

## Open Review 3: Not the Observation of ‘Beautiful’ Forms, But How to Undertake a Theoretical Kaleidoscope of Inequality (Alison MacKenzie)

I have often thought how valuable it would be to look at an issue of injustice from several theoretical or applied philosophical perspectives. In my own master’s teaching, that’s the approach I take but over three modules each one dedicated to the capabilities approach (Nussbaum’s version), epistemic injustice (Fricker 2007), and deconstruction using Bourdieu and Foucault. The students are free to choose any injustice and they are varied. The popular ones are disability, sexual violence and harassment, and medical–endometriosis or the menopause, for example. The students are free to examine the same injustice in each module in order to develop a deep understanding of the varieties of injustice, how entrenched and invisible they can be, and why. Injustice serves some people very well. They enjoy privilege, status, wealth, and power. The privileged have many means to keep the injustice alive or obscured: subjugation, suppression, denigration, threats, and so on. So, I was intrigued when Petar asked me to review this article.

I like the approach and I like the idea of a kaleidoscope. I had one as a child. I recall my endless fascination of, and absorption in, the changing colours and shapes. I thought it was beautiful and mysterious. Kaleidoscope is formed from three Greek words–*kalos* which means beautiful, *eidos* which means form, and *scopeo* which means to examine–and denotes the observation of beautiful forms. As a concept, it is charming, denoting childlike curiosity at how the world can change just by looking down the eyepiece and rotating the cylinder to observe an infinite variety of forms shift into view.

‘Kaleidoscope’ might, then, seem an odd choice of word to describe the authors’ approach because inequality is not charming, it is not beautiful or innocent, and its form changes only insofar as technology or progress finds new ways for inequality to persist. Standardly, we use it to mean ‘constantly changing’ or ‘shifting patterns’. It refers to kaleidoscope of theories. Understanding the ‘supercomplexity’ of inequality, as Kuhn et al. aptly describe it, is not easy, mainly because as researchers we tend to look at inequality one theory at a time or through only one theory over the course of one’s academic life (as Marxists, Foucauldians, capabilitarians, for example). We do need to understand inequality and injustice from multiple perspectives in one place.

What is the best way to do this? Not in articles for journals. The standard wordcount does not permit good quality analysis of injustice and inequality from anything more than one perspective as academics have no taste for theoretical ‘soups’. Even if writers have nearly double the word count, as the authors do here, all they can achieve is a proposition on what could be done, to give the briefest indication of what such an analysis might look like. This also relies on the reader having some background knowledge to appreciate what the kaleidoscopic approach is presenting to our minds. For full-scale treatment, it requires a detailed exposition of the theory and then its application to the issue–and that needs about 8000 words.

But this provides an opportunity if the authors of this article were interested in taking their idea further, a book. A single issue–digital inequalities–explored over eight chapters from eight perspectives, with a final chapter that concludes on the insights of digital inequality from a capabilities approach, critical realism, affective injustice, and so on. This could be a compelling and important contribution to the Postdigital Science and Education book series.^3^

## Footnotes

1 This work is explained in more detail by The Web Foundation. See https://webfoundation.org/research/2022-meaningful-connectivity-report/. Accessed 24 December 2022.

2 Considering that CR is a philosophical approach to social science, it devotes more space that this paper does not have. Thus, we refer the interested reader to look for more information at https://criticalrealismnetwork.org/webinarvideos/. Accessed 18 January 2023.

3 See https://www.springer.com/series/16439. Accessed 3 February 2023.
